# Single Circulating-Tumor-Cell-Targeted Sequencing to Identify Somatic Variants in Liquid Biopsies in Non-Small-Cell Lung Cancer Patients

**DOI:** 10.3390/cimb44020052

**Published:** 2022-02-02

**Authors:** Mouadh Barbirou, Amanda Miller, Yariswamy Manjunath, Arturo B. Ramirez, Nolan G. Ericson, Kevin F. Staveley-O’Carroll, Jonathan B. Mitchem, Wesley C. Warren, Aadel A. Chaudhuri, Yi Huang, Guangfu Li, Peter J. Tonellato, Jussuf T. Kaifi

**Affiliations:** 1 Center for Biomedical Informatics, Department of Health Management and Informatics, School of Medicine, University of Missouri, Columbia, MO 65212, USA; barbiroum@missouri.edu (M.B.); milleraa@health.missouri.edu (A.M.); tonellatop@health.missouri.edu (P.J.T.); 2 Department of Surgery, University of Missouri School of Medicine, Columbia, MO 65212, USA; yariswamym@health.missouri.edu (Y.M.); ocarrollk@health.missouri.edu (K.F.S.-O.); mitchemj@health.missouri.edu (J.B.M.); liguan@health.missouri.edu (G.L.); 3 Harry S. Truman Memorial Veterans’ Hospital, Columbia, MO 65201, USA; 4 RareCyte®, Inc., Seattle, WA 98121, USA; aramirez@rarecyte.com (A.B.R.); nericson@rarecyte.com (N.G.E.); 5 Siteman Cancer Center, St. Louis, MO 63110, USA; warrenwc@missouri.edu (W.C.W.); aadel@wustl.edu (A.A.C.); huangyi1@wustl.edu (Y.H.); 6 Department of Animal Sciences and Surgery, Informatics and Data Sciences Institute, Bond Life Sciences Center, University of Missouri, Columbia, MO 65211, USA; 7 Department of Radiation Oncology, Washington University School of Medicine, St. Louis, MO 63110, USA

**Keywords:** circulating tumor cells, non-small-cell lung cancer, single cell next generation sequencing

## Abstract

Non-small-cell lung cancer (NSCLC) accounts for most cancer-related deaths worldwide. Liquid biopsy by a blood draw to detect circulating tumor cells (CTCs) is a tool for molecular profiling of cancer using single-cell and next-generation sequencing (NGS) technologies. The aim of the study was to identify somatic variants in single CTCs isolated from NSCLC patients by targeted NGS. Thirty-one subjects (20 NSCLC patients, 11 smokers without cancer) were enrolled for blood draws (7.5 mL). CTCs were identified by immunofluorescence, individually retrieved, and DNA-extracted. Targeted NGS was performed to detect somatic variants (single-nucleotide variants (SNVs) and insertions/deletions (Indels)) across 65 oncogenes and tumor suppressor genes. Cancer-associated variants were classified using OncoKB database. NSCLC patients had significantly higher CTC counts than control smokers (*p* = 0.0132; Mann–Whitney test). Analyzing 23 CTCs and 13 white blood cells across seven patients revealed a total of 644 somatic variants that occurred in all CTCs within the same subject, ranging from 1 to 137 per patient. The highest number of variants detected in ≥1 CTC within a patient was 441. A total of 18/65 (27.7%) genes were highly mutated. Mutations with oncogenic impact were identified in functional domains of seven oncogenes/tumor suppressor genes (*NF1, PTCH1, TP53, SMARCB1, SMAD4, KRAS,* and *ERBB2*). Single CTC-targeted NGS detects heterogeneous and shared mutational signatures within and between NSCLC patients. CTC single-cell genomics have potential for integration in NSCLC precision oncology.

## 1. Introduction

Lung cancer is by far the leading cause of cancer-related deaths worldwide. Non-small-cell lung cancer (NSCLC) accounts for >80% of all lung cancer subtypes [1]. Although lung cancer screening of long-term smokers by low-dose computed tomography (LDCT) significantly increases detection at curable stages and improves survival [2], 75% of NSCLC patients are diagnosed at advanced stages III–IV with 5-year survival rates of <25% [3]. The eligibility of NSCLC patients with advanced disease to receive targeted therapy relies on profiling of driver oncogenes and tumor suppressor genes mutation analyses performed on invasive tumor tissue biopsies [4]. However, these tumor tissue biopsies are associated with significant morbidities and costs. Due to these limitations, invasive biopsies are typically only performed once and consequently do not reflect tumor evolution over time and development of resistant clones during therapies [5,6]. Therefore, developing non-invasive and repeatable, real-time diagnostic tests for NSCLC patients appears critical to improve management [7].

Liquid biopsy approaches by a simple blood draw are minimally invasive and easily repeatable alternatives to tissue biopsies [8]. For example, cell-free circulating tumor (ct)DNA has evolved as a blood-based option to identify genetic tumor alterations for NSCLC and other cancer patients [9,10]. Whereas ctDNA release is thought to be related to tumor cell turnover [11,12], circulating tumor cells (CTCs) are shed into the blood from the primary tumor [13] and may be reflective of tumor resistance to treatment. ctDNA is a useful biomarker to predict disease recurrence following surgical tumor resections and is a predictor of treatment responses in solid cancers, such as in patients suffering from malignant melanoma [14]. CTC-derived DNA may also offer mutational insights into future metastatic recurrences, as CTCs represent a whole cancer and—in some cases—exhibit tumorigenic properties [15]. Importantly, recent studies suggest that CTCs exhibit unique genetic alterations that are not detected in ctDNA, whereas ctDNA can reveal genomic alterations not detected in CTCs [16]. These findings of unique mutations detected by different liquid biopsy modalities provide a strong rationale for further exploration of CTC single-cell sequencing assays. Novel CTC sequencing liquid biomarker assays are likely to provide novel information for NSCLC patients to investigate resistance mechanisms for personalized medicine [8,17].

In this study, a CTC detection platform that integrates detection and single-cell retrieval for targeted NGS of individual CTCs was applied to NSCLC patients. In a prospective pilot trial, CTCs were enumerated and single CTCs (and control white blood cells (WBCs)) underwent targeted NGS to detect somatic variants in oncogenes and tumor suppressor genes in liquid biopsies. To serve as risk-matched controls, long-term smokers without lung cancer were recruited from a lung cancer screening program. Single CTC-targeted NGS could detect heterogeneous and shared mutational signatures within and between NSCLC patients. In addition to other liquid biomarkers, CTC single-cell genomics have potential for integration in NSCLC precision oncology.

## 2. Materials and Methods

### 2.1. Enrollment of Subjects

Subjects were prospectively recruited at the Ellis Fischel Cancer Center at the University of Missouri (MU), consisting of 20 patients with pathologically confirmed NSCLC and 11 high-risk chronic smokers without cancer determined by screening LDCT. Clinicopathologic data were obtained from chart review. The TNM staging manual of the American Joint Committee on Cancer (AJCC, Chicago, IL, USA; 8th edition) was applied. A healthy volunteer blood donor was included for validation of the platform by spiking with a known number of human NSCLC adenocarcinoma cell line A549 cells. Studies involving human subjects were approved by the University of Missouri Institutional Review Board (MU IRB Number 2010166; approved 16 April 2016) and were performed according to the Helsinki Declaration.

### 2.2. CTC Enumeration with NSCLC Cell Line Cells Spiked into Healthy Human Blood and Study Subjects’ Blood Samples

**Figure 1:**
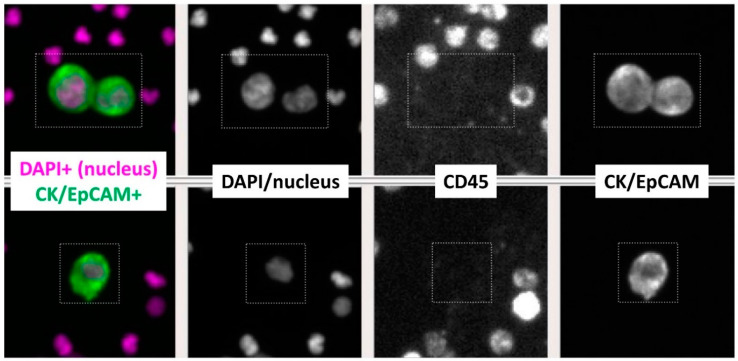
Four-channel fluorescent images of circulating tumor cells (CTCs) detected in NSCLC patients’ blood (7.5 mL). CTCs from two different NSCLC patients are shown, identified as cytokeratin (CK)/EpCAM+ and CD45- cells with DAPI+ nuclei. (Magnification ×10).

In alignment with the traditional definition of a CTC of the FDA-approved CellSearch^®^ platform, a multi-parameter immunofluorescence staining pattern analysis was performed and identified a CTC as CK/EpCAM+ (epithelial markers) and CD45- (WBC marker) with a DAPI+ nucleus (Supplementary Figure S1A). The mean fluorescent intensity values of the whole cell were used to distinguish strong from weak staining for each biomarker. A comparison of multiple slides of cancer cells and WBCs showed that tumor cells consistently displayed strong CK/EpCAM, and weak CD45 staining compared with WBCs in the same sample that had weak CK/EpCAM and strong CD45 expression (Figure 1A). Based on the explicit and distinct pattern of tumor cells, a cut-off was used to define tumor cells with mean fluorescent intensity for CK > 500, EpCAM > 100, and CD45 < 100. For initial validation of the technology (AccuCyte; RareCyte, Seattle, WA, USA) [18] before testing clinical samples, blood samples of a healthy control donor were spiked by single-cell micropipetting with a known number (N = 0, 100, 200, 1000) of human NSCLC adenocarcinoma cancer cells (A549; ATCC) with the analytic personnel being blinded. Results showed a similar immunofluorescence staining profile between the reference spiked A549 cell line in healthy donors’ blood and detected CTCs from study subjects (spiked/retrieved: 0/0; 100/76; 200/208; 1000/1223; linear regression r^2^ = 0.999) (Supplementary Figure S1B). Following validation of correlation with a variety of spiked cancer cells, the same protocols were applied to the clinical samples of NSCLC and screening subjects.

Phlebotomies were performed and blood (7.5 mL) was collected in AccuCyte BCT tubes and shipped overnight to RareCyte Inc. (Seattle, WA, USA) for CTC enumeration and single-cell retrieval of CTCs and WBCs in NSCLC patients. Processing was performed using the AccuCyte sample preparation system to isolate nucleated cells and spread them evenly onto SuperFrost^™^ Plus Microscope Slides (Fisherbrand^™^, Fisher Scientific, Hampton, NH, USA). The slides were air-dried at room temperature and banked for later staining (stored at −20 °C). Enumeration and retrieval of CTCs and WBCs were performed using CyteFinder^®^ instrument based on CF405, Sytox Orange, CF647, and QD800 tags to target the Pre-label, Nucleus, CK/EpCAM, and CD45, respectively. Slide images were analyzed by CyteMapper^®^ software. Then, cells were individually retrieved and dispensed in PCR tubes for downstream NGS. CTCs were defined by nuclear size ≥8 μm in diameter, presence of a well-defined and visible cytoplasm, and immunofluorescence staining in the corresponding channels of predicted biomarkers (CK+ and/or EpCAM+, CD45-, DAPI+ nucleus).

### 2.3. Targeted NGS of Single CTCs

For targeted NGS sequencing of single CTCs, the CleanPlex OncoZoom Panel kit (Paragon Genomics, Inc., Hayward, CA, USA) was used, with a modified protocol to sequence single cells. Standard bioinformatic and visualization workflows for dissemination of sequence data were applied. DNA was extracted from a sorted single cell using a Single Cell Lysis Kit (cat. 4458235; Thermo Fisher Scientific, Indianapolis, IN, USA). Six microliters of total reaction volume was used for first-step amplification. To establish the single-cell retrieval protocol, we used human lung cancer cells (A549) spiked into healthy blood. We retrieved three A549 cells and two WBCs from the healthy donor blood. For subsequent analysis of clinical samples, a targeted-genome amplification and sequencing of 2–6 CTCs and 1–2 WBCs per patient was performed covering 601 amplicons in 65 genes. Concentration and quality of prepared libraries were assessed via fragment analysis using the Advanced Analytics High Sensitivity NGS Fragment Analysis Kit (cat. DNF-474-0500; Agilent, Santa Clara, CA, USA). An individual library quality ratio score was determined by dividing the fragment analysis trace concentration (ng/µL) (250–350 bp peak concentration) by the fragment peak concentration (ng/µL) (150–190 bp). The intent was to remove samples with higher concentration of primer dimers and to remove samples with low concentration of library fragments, presenting poor quality with ratio scores less than 1. Passing libraries were denatured, pooled and diluted to a final loading concentration of 1.5 pMol prior to sequencing on the NextSeq 500 system at 2 × 151 bp using the NextSeq Mid Output v2 (300 cycle) kit (cat. 15057939; Illumina, San Diego, CA, USA). FASTQ files were pre-processed for adapter trimming using cutadapt version 1.18 [19] and then assessed using FastQC [20] and MultiQC [21]. The paired-end reads were aligned to the GRCh37 human reference genome with bwa version 0.7.17 [22]. Subsequent analysis was restricted to the targeted regions of the panel; variant calling was performed in the targeted regions of the OncoZoom panel with 100 bp of padding. The resulting BAM files were cleaned using the base quality score recalibration provided by GATK v. 4.1.9.0 [23].

### 2.4. Somatic Variant (SNVs and Indels) Analysis

Somatic single-nucleotide variants (SNVs) and insertions/deletions (Indels) were called using Mutect2 in GATK v. 4.1.9.0 for each subject individually with the gnomAD database as a “germline-resource” from the GATK resource bundle (https://console.cloud.google.com/storage/browser/genomics-public-data/resources/broad/hg38/v0, accessed on 20 January 2021). Multi-sample mode of Mutect2 [24] was run for a joint analysis that included all CTCs to determine the shared variants among the CTCs within a subject. Then, Mutect2 was run for each individual CTC separately to determine variants in ≥1 CTCs within a subject. Initial variant filtering used FilterMutectCalls with default parameters followed by annotation using ANNOVAR allele frequency and gene information [25]. A second filtering according to the Minor Allele Frequency (MAF) ≥1% in the 1000 Genomes [22] and ExAC databases [26] was performed. Additional annotation by RefSeq Gene definition was performed to predict the variant’s genomic region and corresponding gene name [27]. The shared variants in all CTCs across the samples were grouped by gene. The variants in genes shared by at least two subjects were matched with a potential oncogenic impact according to the open access OncoKB database [28] that annotates biological and oncogenic effects and the prognostic significance of somatic molecular alterations, including the ones predictive of drug responses based on US Food and Drug Administration (FDA) labeling. They were visualized in lollipop plots illustrating the genomic position and functional impact of these variants using cBioPortal MutationMapper [29,30].

### 2.5. Statistical Analysis

Statistical analyses were performed with R version 4.0.2 [31] and Prism v8.0.1 (GraphPad Software, San Diego, CA, USA). To compare non-parametric CTC counts between two groups, the Mann–Whitney test was applied. A *p* value of <0.05 considered statistically significant.

## 3. Results

### 3.1. Clinical Characteristics of Subjects

A total of 31 subjects were prospectively enrolled. Out of these, 20 patients were diagnosed with NSCLC and 11 subjects were risk-matched controls consisting of long-term smokers (all ≥30 pack years) without evidence of lung cancer on screening LDCT scans of the chest. Clinical characteristics of all 31 study subjects are described in Table 1. NSCLC patients were staged by AJCC classification as localized/loco-regional disease stage I-III in N = 9 (45%) and metastatic stage IV in N = 11 (55%). No significant differences were observed between the two study groups of cancer patients and controls with regard to relevant clinical parameters, such as age and extent of smoking history (defined by pack years).

### 3.2. CTC Enumeration in NSCLC Patients and Chronic Smokers without Cancer

**Figure 2:**
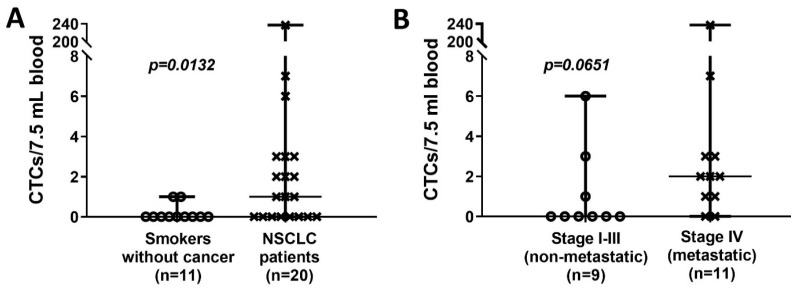
CTC counts in the study populations. (**A**). CTC counts in high-risk controls (long-term smokers) without cancer and patients diagnosed with NSCLC. (**B**). Distribution of CTC count for NSCLC patients by tumor stages, separating them in localized/loco-regional stage I–III versus advanced, metastatic stage IV. (Scatter dot plots; *p* values were calculated with Mann–Whitney test).

A multiplex immunofluorescence approach identified a CTC as CK/EpCAM+ (epithelial markers) and CD45- (WBC marker) with a DAPI+ nucleus (Figure 1). Following validation of protocols and the CTC detection technology in blinded spiking experiments with A549 lung cancer cells into healthy human blood (supplementary Figure S1A,B), CTCs were detected in 12/20 (60%) of NSCLC patients at a mean of 13.4 ± 1.78 SEM with a median of 1 (range 0–237) (Table 2). In long-term smokers without cancer, CTCs were detected in 2/11 (18%) subjects with a mean of 0.18 ± 0.12 SEM and a median of 0 with a range of 0–1. A statistically significantly higher number of CTCs were found in NSCLC patients compared to control long-term smokers without lung cancer as determined by LDCT screening (*p* = 0.0132; Mann–Whitney test) (Figure 2A). Subsequently, we compared the CTC counts between NSCLC patients according to tumor stage, grouping patients into two categories: patients with localized or loco-regional cancer disease (stage I–III) and patients with metastatic disease (stage IV). The mean CTC count was clearly higher in metastatic/stage IV NSCLC patients ((mean 23.45 ± 21.36 SEM; median 2 (range 0–237)) than non-metastatic stage I–III patients ((mean 1.11 ± 0.70 SEM); median 0 (range 0–6)), although not reaching level of statistical significance (*p* = 0.0651) (Figure 2B).

### 3.3. Characterization of Single CTCs Somatic Variants in NSCLC Patients

Seven NSCLC patients (stage I: N = 2; metastatic stage IV: N = 5) that had ≥2 CTCs detected were selected for single-cell sequencing (Table 3). A total of 36 single cells (23 CTCs and 13 WBCs) from these seven NSCLC patients underwent targeted NGS. As we processed an input with low library DNA concentration, only libraries with a library quality ratio score of greater than 1 with clean amplification of the targeted region in the fragment analysis (as defined in the methods) were combined and sequenced after quality-control examination of library DNA concentrations and fragment sizes (Supplementary Figure S2A). With a target of 500-fold coverage, we generated a total of 2,769 Mb data per sample, with a mean of 76.9 and a range of 26 to 129 Mb per sample. A Phred score of 30 for all FASTQ files was observed, indicating high base quality (supplementary Figure S2B).

Somatic variants (the sum of all SNVs and Indels) were counted in sequenced CTCs according to the number of appearances (1) within each subject and (2) across all seven subjects to compare variant incidences and variant types in NSCLC patients. The number of shared variants that were detected in all sequenced CTCs within a subject was determined and is shown in Table 3. Adding up all variants shared by all sequenced CTCs within a subject, a total of 644 shared variants were identified in all seven NSCLC patients combined. After allele frequency filtering using a cutoff of <0.01 MAF based on the 1000 Genomes and ExAC databases and as outlined in the methods, a total of 617 variants remained, and these are summarized per patient in Table 3 (2nd last column). In the two stage I patients, one patient (RL13) had only one variant shared by all CTCs, whereas 85 shared variants were detected in all sequenced CTCs in the other stage I patient (RL5). In the five metastatic/stage IV NSCLC patients, an increased number of variants shared by all CTCs within the subject was found, ranging from 72 to 137.

Finally, variants detected in ≥1 CTCs (but not necessarily in all CTCs) within an NSCLC patient were determined (Table 3; last column). A higher number of variants in ≥1 CTCs within a subject was detected in stage IV/metastatic patients in comparison to the two stage I patients. The highest number of 441 shared variants in ≥1 CTCs was noted within a metastatic NSCLC patient (RL16). In all seven NSCLC patients, the total number of variants detected in ≥1 CTCs within the same subject ranged from 121 to 441.

### 3.4. Single CTC Somatic Variants Detected in Oncogenes and Tumor-Suppressor Genes

Variants detected in the 65 oncogenes and tumor-suppressor genes included in the targeted NGS panel were classified (Table 4). Since some of the 617 shared variants detected in all CTCs within a subjected listed in Table 3 appeared in ≥1 subject, the variants that appeared more than once across the seven patients were counted only once. This reduced the number of shared variants to a total of 598 in various genes. Analysis showed that 18 (27.7%) oncogenes/tumor-suppressor genes were highly mutated, defined as >10 somatic variants detected per gene. Out of these 18 genes, 14 (77.8%) showed a shared somatic variant by at least two patients (Table 5). The highest number of shared variants was detected in the TP53 gene (four variants)—a known tumor-suppressor gene described in multiple cancers, including NSCLC.

**Figure 3:**
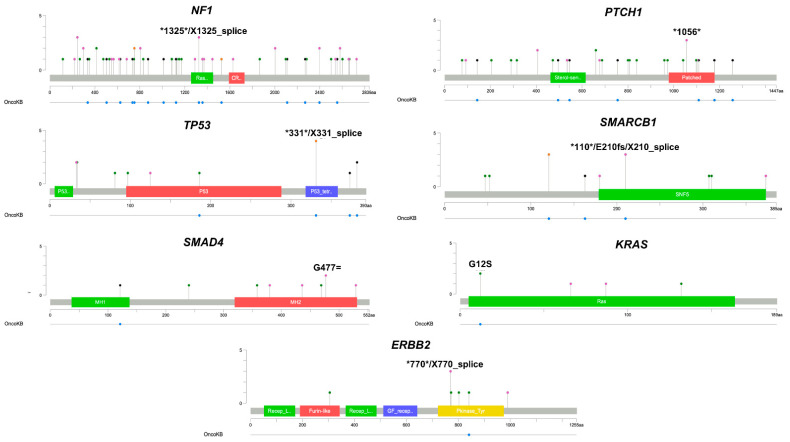
Gene locations of somatic variants in oncogenes and tumor-suppressor genes with predicted oncogenic impact, as per OncoKB database. Seven oncogenes/tumor suppressor genes were identified with shared somatic variants detected in ≥2 CTCs. Lollipop plots show the variant gene locations and their predicted oncogenic impact, according to OncoKB database. In some genes, at the same base position different mutations are observed in multiple individual CTCs. For example, in NF1 we observed a base substitution in coding sequence position of 1325 that results in the introduction of a stop codon and, separately at the same base position, a base substitution occurs that alters the conserved splice acceptor/donor site for exon/intron splicing. Colored boxes represent mutations in specific functional domains (*: stop codon; mutation types: green: missense, black: truncating, orange: splice, pink: others).

Shared somatic variants detected in CTCs among the seven NSCLC patients were also matched against variants described in OncoKB [26], a knowledge base containing somatic mutations in cancer-associated genes with diagnostic and therapeutic relevance. Visualization plots were then generated to highlight genomic alterations and their potential impact on specific functional domains of select genes. Variants in genes with known impact in cancer were found in functional domains in 7 (50%) out to 14 oncogenes/tumor suppressor genes (NF1, PTCH1, TP53, SMARCB1, SMAD4, KRAS, and ERBB2) (Figure 3). With the only exception of PTCH1, 6/7 (85.8%) of these cancer-associated genes have been described to be associated with NSCLC development.

## 4. Discussion

In contrast to invasive lung cancer tissue biopsies, which are associated with significant morbidities and costs, minimally invasive liquid biopsies by simple blood draws hold great promise to improve clinical management of NSCLC. Liquid biopsies in cancer patients can identify somatic variants and cancer-associated mutations at the time of diagnosis or later on in real-time to allow precise adjustments of therapy management or monitoring of disease progression [32]. Beyond CTC enumeration, the present study provides a technical assessment of single CTC-targeted NGS in NSCLC patients. We successfully detected and retrieved single CTCs using 7.5 mL of blood and then performed targeted NGS with a panel targeting more than 2900 hotspots in 65 genes with known cancer-associations. This approach led to identification of distinct and shared variants in and across NSCLC patients’ single CTCs. Cancer-associated variants detected in single CTCs could be matched to known cancer mutations from an established oncology knowledge base. Analysis of a relatively low number of single CTCs per patient still allowed identification of key oncogene and tumor suppressor gene variants known and not yet known to be associated with NSCLC disease.

Current state-of-the-art molecular testing is performed by one-time invasive tumor tissue biopsy. In some cases, low tumor cellularity requires even an invasive repeat biopsy, which is again associated with morbidities, costs, and delay in care. In contrast to non-cellular liquid biomarkers (e.g., ctDNA or extracellular vesicles), a CTC in the blood represents a whole, morphologically intact tumor cell. Lung cancer patient-derived CTCs can be tumorigenic in vivo in immunodeficient mice [15,33], indicating that micrometastatic CTCs may carry mutations of future metastases. In our analysis of single CTCs retrieved from seven NSCLC patients, somatic variants with potential oncologic impact were detected in all CTCs analyzed. CTCs of at least two NSCLC patients shared variants in six oncogenes/tumor suppressor genes: *NF1*, *TP53*, *SMARCB1*, *SMAD4*, *KRAS*, and *ERBB2*. Variants in the *NF1* tumor suppressor gene have been previously described to be present in 10% of NSCLC tumor tissues, and they are frequently paired with *KRAS* and *ERBB* cancer driver variants [34]. Additionally, variants of the *NF1* gene have been found relatively frequently in male smokers and coexist with *TP53* variants [35]. Several studies have reported the *TP53* gene variants as a predictor of NSCLC patients’ poor prognosis [34,36]. For instance, an analysis conducted using The Cancer Genome Atlas (TCGA) revealed that NSCLC patients with *TP53* variants had significantly shorter survival rates than those without [36]. With regard to *SMARCB1*, gene variants and loss of expression were reported in up to 5% of NSCLC cases and have been associated with poor clinical outcome [37]. The *SMAD4* pathway has been identified as a potential target for tumor treatment [38]. The findings of that study indicated that variants of the *SMAD4* gene play an important role for NSCLC metastasis as they were observed in advanced stage IV patients only. *SMAD4* serum concentration also correlated with a malignant NSCLC phenotype that was associated with metastasis and clinical progression [39]. In addition, *SMAD4* and its transcription factor play a regulatory function for many target genes, also increasing the risk of cell tumorigenesis in lung cancer [40]. *SMAD4* variants and related expression may regulate the signal transduction pathways involved in NSCLC tumorigenesis, such as the *TGF-β/SMAD4* pathway [41]. *KRAS* variants were also detected in the current study in single CTCs. *KRAS* variants have been detected in 51% of advanced NSCLC patients, which included older patients and current or former smokers [42]. This study also showed a higher prevalence of 51% of KRAS variants in NSCLC adenocarcinoma patients, in contrast to previously reported prevalence of 20–40% [42]. We also detected variants in the *ERBB2* gene in single CTCs in our cohort, a well-known driver oncogene in NSCLC [43]. *ERBB2* gene is a member of the *EGFR* family that is involved in several biological scenarios in malignant diseases [44]. It has been demonstrated that *ERBB2* is involved in a series of cancer-associated processes, such as cell proliferation, cell survival, and differentiation [45]. With regard to these oncogenes and tumor suppressor genes, previously published results in NSCLC are in concordance with our findings on cancer-associated genes that were found to be mutated in single CTCs of NSCLC patients. Findings support that single CTCs identified using the platform in our study with criteria also applied by the FDA-approved CellSearch^®^ system can be analyzed individually by targeted NGS to identify variants in known NSCLC-associated oncogenes and tumor suppressor genes. As a liquid biopsy modality, single CTC-seq analyses may provide a complementary technology to assist clinicians for better management of NSCLC patients using a non-invasive protocol with a small sample of peripheral blood.

There are several limitations with the present pilot study. Most important is that the cohort is a small sample size, so the analysis has limited power. The study lacks mutational information on the matched tumor tissues that were not analyzed for comparison with the targeted sequencing performed on single CTCs. Matched-tumor tissues for comparative analyses were not available in our study. Additionally, we performed testing just at one timepoint, which did not allow us to study longitudinal changes of detected somatic variants over time. Longer follow-up times, particularly in screening subjects without cancer, and our focus on initial diagnosis resulted in determining mutations at a single time point only. Finally, we did not compare CTC-seq data with other genetic liquid biopsy modalities, such as ctDNA, to correlate findings on CTCs with other liquid biopsy technologies. In particular the integration of CTC-seq with ctDNA findings promises more comprehensive profiling of NSCLC-associated mutations by liquid biopsy.

In summary, our study presents a robust method of CTC detection in NSCLC patients and, as a critical addition, an option for genetic profiling of single CTCs. Distinct and shared variants within and across NSCLC patients can be identified in oncogenes and tumor suppressor genes in single CTCs, even in cases with low CTC numbers. Further investigations, including a larger prospective cohort of different tumor stages of NSCLC patients, will be required for further validation. Single CTC variant detection by sequencing may have potential clinical value for diagnosis and therapy management of NSCLC patients.

## Figures and Tables

**Table 1:** Subjects’ characteristics.

	*N*
Total number of of subjects	31
NSCLC patients	20 (65%)
Median age (range)	66 (55–75)
Gender Females Males	14 (70%) 6 (30%)
Smoking history Never-smokers Smokers Pack years <5 Pack years ≥5–30 Pack years ≥30	3 (15%) 17 (85%) 1 (6%) 2 (12%) 14 (82%)
Tumor stage (TNM/AJCC 8th ed.) I–III (localized/locoregional disease) IV (metastatic)	9 (45%) 11 (55%)
Histologic subtype Adenocarcinoma Squamous cell carcinoma	13 (65%) 7 (35%)
Smokers without cancer	11 (35%)
Median age (range)	67 (52–76)
Gender Females (%) Males (%)	7 (64%) 4 (36%)
Smoking history Pack years ≥30	11 (100%)

**Table 2:** CTC enumeration in control high-risk subjects without cancer and NSCLC patients.

	*N*	Circulating Tumor Cells/7.5 mL Blood			*p* Value
		Present (%)	Mean (±SEM)	Median (Range)	
Smokers without cancer	11	2 (18%)	0.18 (±0.12)	0 (0–1)	
NSCLC patients	20	12 (60%)	13.40 (± 11.78)	1 (0–237)	0.0132 *
NSCLC tumor stage I–III (non-metastatic) IV (metastatic)	9 11	3 (33%) 9 (82%)	1.11 (±0.70) 23.45 (±21.36)	0 (0–6) 2 (0–237)	0.0651 ^†^

* Comparing smokers without cancer vs. NSCLC, ^†^ comparing stage I–III vs. IV: Mann–Whitney test.

**Table 3:** NSCLC patients (N = 7) selected for single CTC sequencing.

Patient ID	NSCLC Stage	Number of CTCs Detected	Number of CTCs Sequenced	Number of WBCs Sequenced	Total Number of Shared Variants Detected in All Sequenced CTCs within the Same Subject	Total Number of Variants Detected in ≥1 Sequenced CTC within the Same Subject
RL13	I	6	4	2	1	125
RL5	I	3	2	1	85	148
RL14	IV	3	3	2	72	121
RL16	IV	237	6	2	130	441
RL17	IV	7	3	2	91	147
RL19	IV	2	2	2	137	194
RL20	IV	3	3	2	101	245

CTC: circulating tumor cells, WBCs: white blood cells.

**Table 4:** Variants per oncogene/tumor-suppressor gene that were detected in all sequenced CTCs within a subject (total number of variants per gene were combined from all seven subjects; variants detected in ≥1 subject were counted once only, adding up to a total of 598 variants).

Gene	Number of Variants
*NF1*	69
*BRCA2*	33
*PTCH1*	33
*NF2*	27
*ATM*	25
*EGFR*	24
*ERBB3*	24
*PIK3CA*	19
*APC*	17
*BRCA1*	17
*RB1*	13
*SMARCB1*	13
*TP53*	13
*CDH1*	12
*CSF1R*	11
*NOTCH1*	11
*SMO*	11
*TERT*	11
*ABL1*	10
*MLH1*	10
*EZH2*	9
*FGFR2*	9
*SMAD4*	9
*DNMT3A*	8
*FBXW7*	8
*MTOR*	8
*CTNNB1*	7
*ERBB2*	7
*HNF1A*	7
*KIT*	7
*PIK3R1*	7
*PTEN*	7
*ALK*	6
*CDKN2A*	6
*PTPN11*	6
*ERBB4*	5
*JAK2*	5
*JAK3*	5
*RET*	5
*BRAF*	4
*KRAS*	4
*LOC100507346*	4
*STK11*	4
*VHL*	4
*FGFR3*	3
*FLT3*	3
*HRAS*	3
*IDH1*	3
*IDH2*	3
*KDR*	3
*MET*	3
*NPM1*	3
*TSC1*	3
*AKT1*	2
*FGFR1*	2
*GNAQ*	2
*GNAS*	2
*NRAS*	2
*PDGFRA*	2
*ATM; C11orf65*	1
*FBXW7-AS1*	1
*GNA11*	1
*MAP2K1*	1
*MSH6*	1

**Table 5:** Variants detected in oncogenes/tumor-suppressor genes in all sequenced CTCs within a subject that were shared by ≥2 NSCLC patients.

Gene	Number of Variants
*TP53*	4
*NF1*	2
*SMARCB1*	1
*SMAD4*	1
*PTEN*	1
*PTCH1*	1
*MAP2K1*	1
*KRAS*	1
*JAK3*	1
*ERBB2*	1
*DNMT3A*	1
*CTNNB1*	1
*ABL1*	1
*ALK*	1

## Data Availability

The data presented in this study are available on request from the corresponding author.

## Supplementary Materials

The following supporting information can be downloaded at: https://www.mdpi.com/article/10.3390/cimb44020052/s1.
