# Supplementary material for: Single Circulating-Tumor-Cell-Targeted Sequencing to Identify Somatic Variants in Liquid Biopsies in Non-Small-Cell Lung Cancer Patients
Source: Curr Issues Mol Biol. 2022 Feb 2;44(2):750–63. doi: 10.3390/cimb44020052 (PMC8928994; doi:10.3390/cimb44020052)
Supplement: Supplementary file 1 [file cimb-44-00052-s001.zip › cimb-1573507-supplementary.pdf]

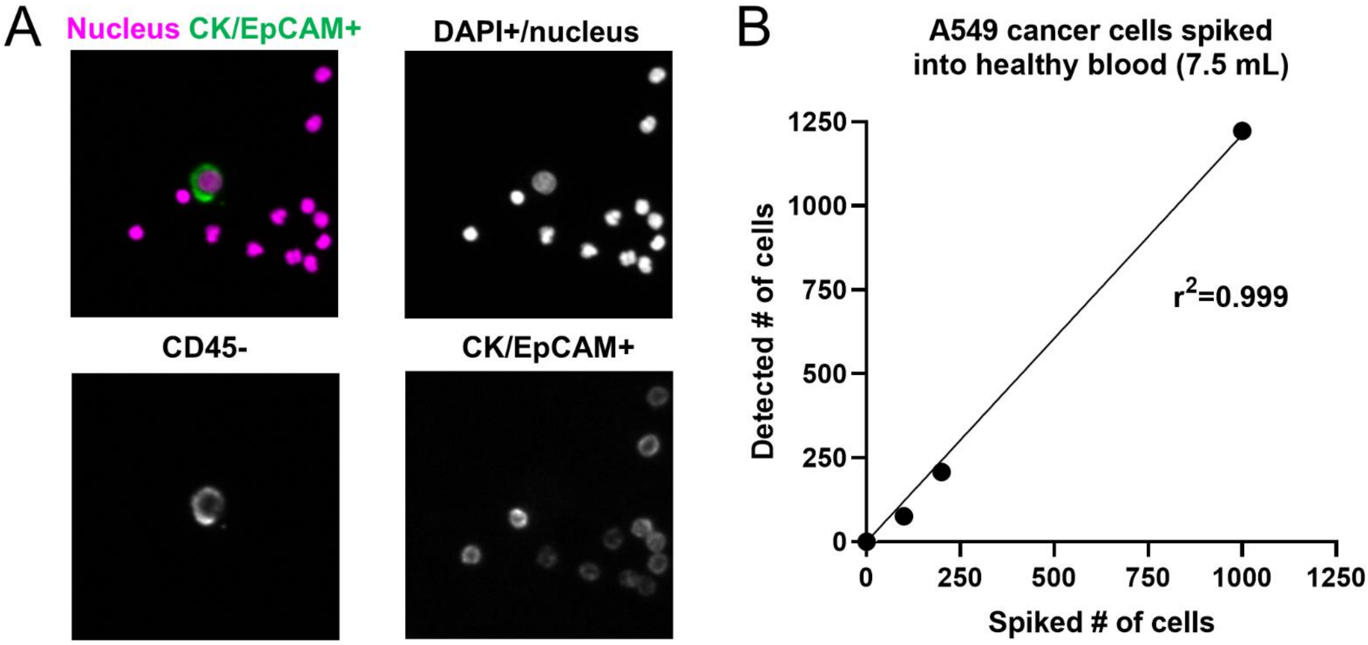

Figure S1. Validation of the CTC detection technology with A549 lung cancer cell-line spiking experiments. (**A**) Representative 4-channel fluorescent images of single human lung cancer cells (A549) that were spiked into healthy human blood for validation of the applied CTC technology. (**B**) Linear regression analysis demonstrating correlation of spiked versus detected lung cancer cell numbers (spiked = 0, detected = 0; 100, 76; 200, 208; 1000, 1223).

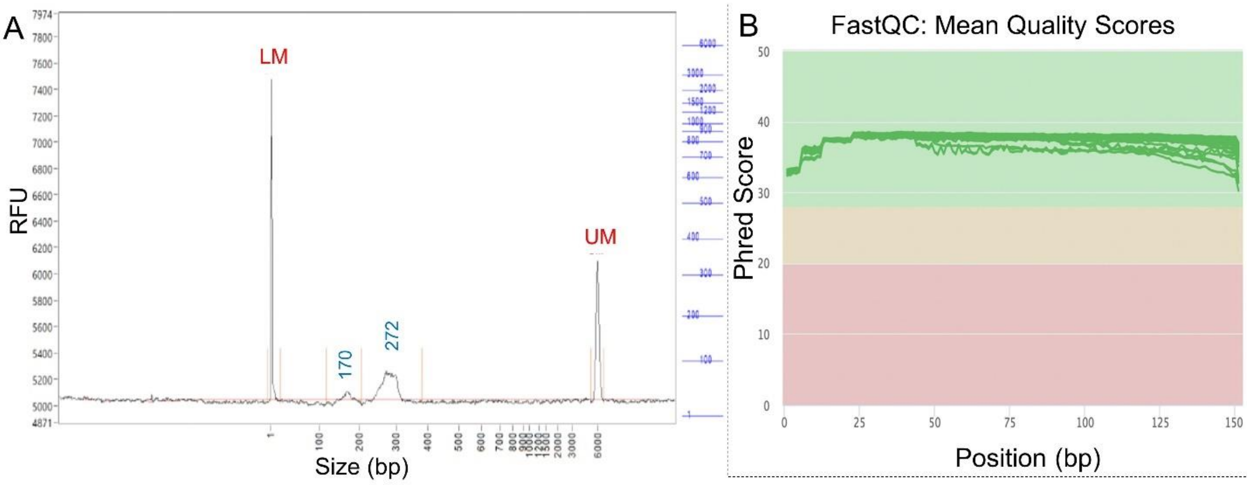

Peak	Size (bp)	Conc. (ng/µl)	From (bp)	To (bp)	Avg. Size (bp)	CV (%)	RFU	Corrected Peak Area
1	1 (LM)	0.0132	0	19	1	168.56	2437	13.178
2	170	0.0162	116	204	168	4.60	58	1.349
3	272	0.1288	204	381	277	6.06	214	10.733
4	6000 (UM)	0.0074	5224	6719	5984	2.80	1057	7.357

TIC (ng/µl)	0.1449
TIM (nmole/L)	0.9229
Total Conc. (ng/µl)	0.1458

Figure S2. Quality control (QC) analysis of the pooled library and the generated sequences. (**A**): DNA concentrations and fragment sizes of the pooled library. (**B**): FASTQC and MultiQC of the generated FASTQ files. (bp: pair of bases, 170 bp: Primer dimers, 272 bp: Pooled libraries amplicon).
